# Supplementary material for: Characterization of Bacteriophages against Pseudomonas Syringae pv. Actinidiae with Potential Use as Natural Antimicrobials in Kiwifruit Plants
Source: Microorganisms. 2020 Jun 29;8(7):974. doi: 10.3390/microorganisms8070974 (PMC7409275; doi:10.3390/microorganisms8070974)
Supplement: Supplementary file 1 [file microorganisms-08-00974-s001.pdf]

Supplementary Table 1. Susceptibility of Chilean Psa isolates to Chilean Psa phages. The susceptibility of 18 different Psa isolates to the different isolated phages was determined through spot test and double-agar assay. Susceptibility level was determined according to the lytic plaque morphology; +++, Strong Activity (Complete clear lysis plaque); ++, Medium activity (Clear throughout but with faintly hazy background lysis plaque); +, Light activity (Turbid lysis plaque); -, No activity (none lysis plaque observed).

**Phages**	**Psa isolates**																	
	159	889	510	429	387	771	233	743	137	817	381	394	144	386	598	784	882	189
CHF1	+++	+++	+++	+++	+++	+++	+++	+++	+++	+++	+++	+++	+++	+++	++	++	++	++
CHF2	+++	+++	+++	+++	+++	+++	+++	+++	+++	+++	+++	+++	+++	+++	++	+++	++	++
CHF3	+++	+++	+++	+++	+++	+++	+++	+++	+++	+++	+++	+++	+++	+++	++	++	++	++
CHF4	+++	+++	+++	+++	+++	+++	+++	+++	++	++	+++	+++	+++	+++	+++	+++	++	++
CHF7	+++	+++	+++	+++	+++	+++	+++	++	+++	++	+++	+++	+++	+++	+++	+++	++	++
CHF9	+++	+++	+++	+++	+++	+++	+++	+++	+++	+++	+++	+++	++	+++	+++	+++	+++	+++
CHF10	+++	+++	+++	+++	+++	+++	+++	+++	+++	+++	+++	+++	++	+++	+++	+++	+++	+++
CHF15	+++	+++	+++	+++	+++	+++	+++	+++	+++	+++	+++	+++	+++	+++	+++	++	+++	+++
CHF16	+++	+++	+++	+++	+++	+++	+++	+++	+++	+++	+++	+++	+++	+++	+++	++	+++	+++
CHF17	+++	+++	+++	+++	+++	+++	+++	+++	+++	+++	+++	+++	+++	+++	+++	++	+++	+++
CHF18	+++	+++	+++	+++	+++	+++	+++	+++	+++	+++	+++	+++	+++	+++	+++	++	+++	+++
CHF19	+++	+++	+++	+++	+++	+++	+++	+++	+++	+++	+++	+++	+++	+++	+++	++	+++	+++
CHF21	+++	+++	+++	+++	+++	+++	+++	+++	+++	+++	+++	+++	+++	+++	+++	+++	+++	+++
CHF30	+++	+++	+++	+++	+++	+++	+++	+++	+++	+++	+++	+++	+++	+++	+++	+++	+++	+++
CHF33	+++	+++	+++	+++	+++	+++	+++	+++	+++	+++	+++	+++	+++	+++	+++	+++	+++	+++

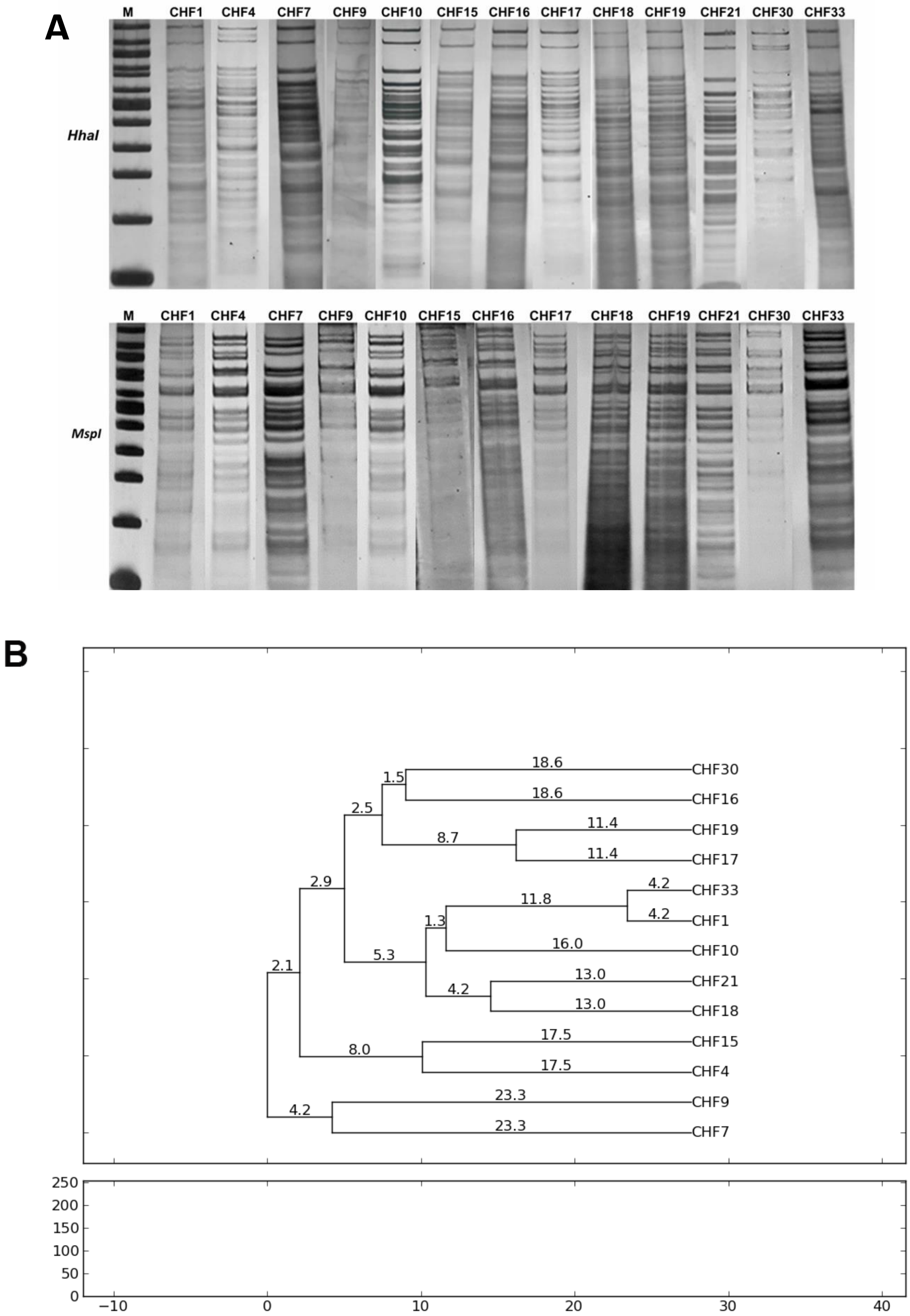
A
M CHF1 CHF4 CHF7 CHF9 CHF10 CHF15 CHF16 CHF17 CHF18 CHF19 CHF21 CHF30 CHF33
HhaI
MspI
B
18.6
CHF30
18.6
CHF16
1.5
2.5
8.7
11.4
CHF19
11.4
CHF17
2.9
4.2
CHF33
4.2
CHF1
11.8
1.3
16.0
CHF10
5.3
2.1
13.0
CHF21
4.2
13.0
CHF18
17.5
CHF15
8.0
17.5
CHF4
23.3
CHF9
4.2
23.3
CHF7
250
200
150
100
50
0
−10
0
10
20
30
40

C

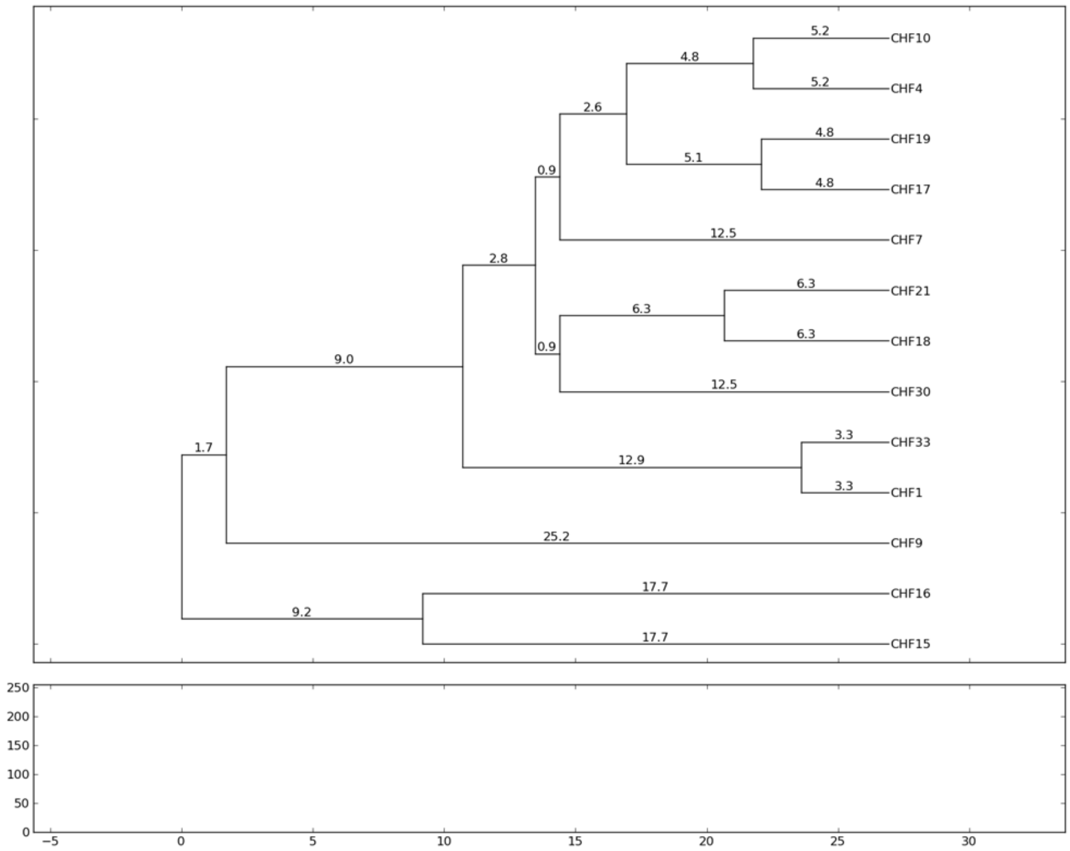

Supplementary Figure 1: RFLP analysis of bacteriophage genomes. (A) Composed image of Genomic DNA of bacteriophages digested with the enzymes *HhaI* and *MspI*. Restriction patterns were separated and visualized by acrylamide:bis-acrylamide gels stained with silver nitrate. The name of the bacteriophages is indicated in the center of the figure. M: Molecular weight marker 100 bp (NEB). **(B)** Restriction pattern analysis of phage genomes digested with *HhaI* using the software PyElph. **(C)** Restriction pattern analysis of phage genomes digested with *MspI* using the software PyElph.

Supplementary Table 2. Genomic characteristics of Chilean Psa phages

Phage	Genome size (pb)	%GC	ORFs[a]	Genus	Identity (%) phiPSA2[b]
CHF1	40.999	57,3	49	T7-like	94.0 %
CHF7	40.557	57,4	48	T7-like	96.4 %
CHF17	40.882	57,3	48	T7-like	93.2%
CHF19	40.882	57,3	48	T7-like	93.2 %
CHF21	40.557	57,4	48	T7-like	93.8 %
CHF33	40.999	57,3	49	T7-like	94.0 %

a. Number of ORFs founded in each genome.
b. Psa phage genomes were aligned with phiPsa2 using Mauve.

Supplementary Table 3. Genome identity (%) between selected Chilean Psa phages.

Phage[a]	CHF1	CHF7	CHF17	CHF19	CHF21
**CHF1**					
**CHF7**	96.0				
**CHF17**	96.4	96.1			
**CHF19**	96.4	96.1	99.99		
**CHF21**	95.4	98.2	95.4	95.4	
**CHF33**	99.96	96.0	96.4	96.4	95.3

a. Access number Genbank (NCBI): CHF1 (MN729595); CHF7 (MN729596); CHF17 (MN729600); CHF19 (MN729597); CHF21 (MN729598); CHF33 (MN729599).

Supplementary Table 4. Annotation of Psa Bacteriophages. ORFs identified for each genome of Psa bacteriophages were annotated according to Blasp analysis. Access number of phages genomes are indicated (GenBank NCBI).

vB_PsaP-CHF 1 (MN729595)

ORF	Annotation	Protein Size (aa)	Identity with first Blastp hit protein (%)	Putative Function of first Blastp hit protein (Access number, NCBI)
0	Hypothetical protein HP0	100	44	Hypothetical protein PsPphi15_gp01 [Pseudomonas phage phi15] (YP_004286179.1)
1	Hypothetical protein HP1	84	90	Hypothetical protein WRT_001 [Pseudomonas virus WRT] (ARM69565.1)
2	Hypothetical protein HP2	66	65	Hypothetical protein PsPhHenninger_gp37 [Pseudomonas phage Henninger] (AUV61710.1)
3	Hypothetical protein HP3	103	93	Hypothetical protein PsPhHenninger_gp36 [Pseudomonas phage Henninger] (AUV61711.1)
4	Hypothetical protein HP4	159	99	Hypothetical protein phiPSA2_03 [Pseudomonas phage phiPSA2] (YP_009043231.1)
5	Hypothetical protein HP5	178	100	Hypothetical protein phiPSA2_04 [Pseudomonas phage phiPSA2] (YP_009043232.1)
6	Hypothetical protein HP6	234	100	Hypothetical protein phiPSA2_05 [Pseudomonas phage phiPSA2] (YP_009043233.1)
7	Hypothetical protein HP7	86	41	Hypothetical protein PsPhHenninger_gp32 [Pseudomonas phage Henninger] (AUV61715.1)
8	Hypothetical protein HP8	144	99	Hypothetical protein VO98_045 [Pseudomonas phage phiPsa17] (AKG94351.1)
9	RNA polymerase	885	100	T3/T7-like RNA polymerase [Pseudomonas phage phiPSA2] (YP_009043235.1)

10	Hypothetical protein HP9	138	45	HNH endonuclease [Pseudomonas fluorescens] (WP_080547111.1)
11	Hypothetical protein HP10	124	88	Hypothetical protein gh-1p04 [Pseudomonad phage gh-1] (NP_813750.1)
12	DNA ligase	354	99	DNA ligase [Pseudomonas phage phiPsa17] (AKG94356.1)
13	Hypothetical protein HP11	93	99	Hypothetical protein phiPSA2_12 [Pseudomonas phage phiPSA2] (YP_009043240.1)
14	Hypothetical protein HP12	85	100	Hypothetical protein phiPSA2_13 [Pseudomonas phage phiPSA2] (YP_009043241.1)
15	Hypothetical protein HP13	215	99	Hypothetical protein gh-1p07 [Pseudomonad phage gh-1] (NP_813753.1)
16	RNA inhibitor	55	100	Putative host RNA-polymerase inhibitor [Pseudomonad phage gh-1] (NP_813754.1)
17	Hypothetical protein HP14	121	100	Hypothetical protein phiPSA2_16 [Pseudomonas phage phiPSA2] (YP_009043244.1)
18	ssDNA binding protein	233	100	Single-stranded DNA-binding protein [Pseudomonad phage gh-1] (NP_813756.1)
19	Endonuclease	147	100	Endonuclease I [Pseudomonad phage gh-1] (NP_813757.1)
20	Lyzozyme	146	100	Lysozyme [Pseudomonad phage gh-1] (NP_813758.1)
21	Hypothetical protein HP15	179	99	Hypothetical protein phiPSA2_20 [Pseudomonas phage phiPSA2] (YP_009043248.1)
22	Primase/Helicase	563	99	Primase/helicase protein [Pseudomonas phage phiPsa17] (AKG94365.1)
23	Hypothetical protein HP16	67	99	Hypothetical protein phiPSA2_22 [Pseudomonas phage phiPSA2] (YP_009043250.1)

24	Hypothetical protein HP17	169	100	Hypothetical protein gh-1p17 [Pseudomonad phage gh-1] (NP_813763.1)
25	DNA polymerase	715	99	DNA polymerase [Pseudomonas phage phiPsa17] (AKG94368.1)
26	Hypothetical protein HP18	127	76	Hypothetical protein VO98_135 [Pseudomonas phage phiPsa17] (AKG94369.1)
27	Hypothetical protein HP19	69	100	Hypothetical protein gh-1p20 [Pseudomonad phage gh-1] (NP_813766.1)
28	Exonuclease	314	100	Exonuclease [Pseudomonad phage gh-1] (NP_813767.1)
29	Hypothetical protein HP20	80	100	Hypothetical protein gh-1p22 [Pseudomonad phage gh-1] (NP_813768.1)
30	Hypothetical protein HP21	90	100	Hypothetical protein phiPSA2_29 [Pseudomonas phage phiPSA2] (YP_009043257.1)
31	Hypothetical protein HP22	147	100	Hypothetical protein phiPSA2_30 [Pseudomonas phage phiPSA2] (YP_009043258.1)
32	Tail assembly	100	100	Tail assembly protein [Pseudomonas phage phiPSA2] (YP_009043259.1)
33	Head-tail connector protein	543	99	Head-tail connector protein [Pseudomonad phage gh-1] (NP_813772.1)
34	Capsid assembly protein	291	100	Capsid assembly protein [Pseudomonad phage gh-1] (NP_813773.1)
35	Major capsid protein	347	100	Major capsid protein [Pseudomonad phage gh-1] (NP_813774.1)
36	Tail tubular protein A	195	99	Tail tubular protein A [Pseudomonad phage gh-1] (NP_813775.1)
37	Tail fiber protein	718	99	Tail fiber protein [Pseudomonas phage phiPsa17]

				(AKG94380.1)
38	Internal virion protein A	144	100	Internal virion protein A [Pseudomonas phage phiPSA2] (YP_009043265.1)
39	Internal virion protein B	193	100	Internal virion protein B [Pseudomonad phage gh-1] (NP_813778.1)
40	Internal virion C	738	100	Internal virion protein C [Pseudomonas phage phiPSA2] (YP_009043267.1)
41	Internal virion D	1392	99	Internal virion protein D [Pseudomonad phage gh-1] (NP_813780.1)
42	Tail fiber protein	609	99	Tail fiber protein [Pseudomonas phage phiPSA2] (YP_009043269.1)
43	Hypothetical protein HP23	118	100	Hypothetical protein phiPSA2_42 [Pseudomonas phage phiPSA2] (YP_009043270.1)
44	Holin	71	100	Type II holing [Pseudomonas phage phiPSA2] (YP_009043271.1)
45	DNA packaging protein	85	99	DNA packaging protein, small subunit [Pseudomonad phage gh-1] (NP_813784.1)
46	Lysin	149	100	Rz-like lysis protein [Pseudomonas phage phiPSA2] (YP_009043273.1)
47	DNA packaging protein B	581	99	DNA packaging protein B [Pseudomonad phage gh-1] (NP_813786.1)
48	Hypothetical protein HP24	57	100	Hypothetical protein gh-1p42 [Pseudomonad phage gh-1] (NP_813788.1)

vB_PsaP-CHF 7 (MN729596)

ORF	Annotation	Protein Size (aa)	Identity with first Blastp hit protein (%)	Putative Function of first Blastp hit protein (Access number, NCBI)

1	Hypothetical protein HP1	84	99	Hypothetical protein WRT_001 [Pseudomonas virus WRT] (ARM69565.1)
2	Hypothetical protein HP2	66	65	Hypothetical protein PsPhHenninger_gp37 [Pseudomonas phage Henninger] (AUV61710.1)
3	Hypothetical protein HP3	103	93	Hypothetical protein PsPhHenninger_gp36 [Pseudomonas phage Henninger] (AUV61711.1)
4	Hypothetical protein HP4	159	99	Hypothetical protein phiPSA2_03 [Pseudomonas phage phiPSA2] (YP_009043231.1)
5	Hypothetical protein HP5	178	100	Hypothetical protein phiPSA2_04 [Pseudomonas phage phiPSA2] (YP_009043232.1)
6	Hypothetical protein HP6	234	99	Hypothetical protein phiPSA2_05 [Pseudomonas phage phiPSA2] (YP_009043233.1)
**7**	Hypothetical protein HP7	86	40	Hypothetical protein PsPhHenninger_gp32 [Pseudomonas phage Henninger] (AUV61715.1)
8	Hypothetical protein HP8	140	100	Hypothetical protein VO98_045 [Pseudomonas phage phiPsa17] (AKG94351.1)
9	RNA polymerase	886	99	DNA-directed RNA polymerase [Pseudomonas phage phiPsa17] (AKG94352.1)
10	Hypothetical protein HP9	90	100	Hypothetical protein phiPSA2_09 [Pseudomonas phage phiPSA2] (YP_009043237.1)
11	Hypothetical protein HP10	128	98	Hypothetical protein VO98_065 [Pseudomonas phage phiPsa17] (AKG94355.1)
12	DNA ligase	354	99	DNA ligase [Pseudomonas phage phiPsa17] (AKG94356.1)
13	Hypothetical protein HP11	93	99	Hypothetical protein phiPSA2_12 [Pseudomonas phage phiPSA2] (YP_009043240.1)

14	Hypothetical protein HP12	85	100	Hypothetical protein phiPSA2_13 [Pseudomonas phage phiPSA2] (YP_009043241.1)
15	Hypothetical protein HP13	215	99	Hypothetical protein gh-1p07 [Pseudomonad phage gh-1] (NP_813753.1)
16	RNA inhibitor	55	100	Putative host RNA-polymerase inhibitor [Pseudomonad phage gh-1] (NP_813754.1)
17	Hypothetical protein HP14	121	100	Hypothetical protein phiPSA2_16 [Pseudomonas phage phiPSA2] (YP_009043244.1)
18	ssDNA binding protein	233	100	Single-stranded DNA-binding protein [Pseudomonad phage gh-1] (NP_813756.1)
19	Endonuclease	147	100	Endonuclease I [Pseudomonad phage gh-1] (NP_813757.1)
20	Lyzozyme	146	100	Lysozyme [Pseudomonad phage gh-1] (NP_813758.1)
21	Hypothetical protein HP15	179	99	Hypothetical protein phiPSA2_20 [Pseudomonas phage phiPSA2] (YP_009043248.1)
22	Primase/Helicase	563	99	Primase/helicase protein [Pseudomonas phage phiPsa17] (AKG94365.1)
23	Hypothetical protein HP16	67	100	Hypothetical protein phiPSA2_22 [Pseudomonas phage phiPSA2] (YP_009043250.1)
24	Hypothetical protein HP17	169	99	Hypothetical protein gh-1p17 [Pseudomonad phage gh-1] (NP_813763.1)
25	DNA polymerase	715	99	DNA polymerase [Pseudomonas phage phiPsa17] (AKG94368.1)
26	Hypothetical protein HP18	127	100	Hypothetical protein gh-1p19 [Pseudomonad phage gh-1] (NP_813765.1)
27	Hypothetical protein HP19	69	98	Hypothetical protein gh-1p20 [Pseudomonad phage gh-1] (NP_813766.1)
				Exonuclease

28	Exonuclease	314	100	[Pseudomonad phage gh-1] (NP_813767.1)
29	Hypothetical protein HP20	80	100	Hypothetical protein gh-1p22 [Pseudomonad phage gh-1] (NP_813768.1)
30	Hypothetical protein HP21	90	100	Hypothetical protein phiPSA2_29 [Pseudomonas phage phiPSA2] (YP_009043257.1)
31	Hypothetical protein HP22	147	100	Hypothetical protein phiPSA2_30 [Pseudomonas phage phiPSA2] (YP_009043258.1)
32	Tail assembly	100	100	Tail assembly protein [Pseudomonas phage phiPSA2] (YP_009043259.1)
33	Head-tail connector protein	543	99	Head-tail connector protein [Pseudomonad phage gh-1] (NP_813772.1)
34	Capsid assembly protein	291	100	Capsid assembly protein [Pseudomonad phage gh-1] (NP_813773.1)
35	Major capsid protein	347	100	Major capsid protein [Pseudomonad phage gh-1] (NP_813774.1)
36	Tail tubular protein A	195	99	Tail tubular protein A [Pseudomonad phage gh-1] (NP_813775.1)
37	Tail fiber protein	718	99	Tail fiber protein [Pseudomonas phage phiPsa17] (AKG94380.1)
38	Internal virion protein A	144	100	Internal virion protein A [Pseudomonas phage phiPSA2] (YP_009043265.1)
39	Internal virion protein B	193	100	Internal virion protein B [Pseudomonad phage gh-1] (NP_813778.1)
40	Internal virion C	738	100	Internal virion protein C [Pseudomonas phage phiPSA2] (YP_009043267.1)
41	Internal virion D	1392	99	Internal virion protein D [Pseudomonad phage gh-1] (NP_813780.1)

42	Tail fiber protein	609	99	Tail fiber protein [Pseudomonas phage phiPSA2] (YP_009043269.1)
43	Hypothetical protein HP23	118	100	Hypothetical protein phiPSA2_42 [Pseudomonas phage phiPSA2] (YP_009043270.1)
44	Holin	71	100	Type II holing [Pseudomonas phage phiPSA2] (YP_009043271.1)
45	DNA packaging protein	85	99	DNA packaging protein, small subunit [Pseudomonad phage gh-1] (NP_813784.1)
46	Lysin	149	100	Rz-like lysis protein [Pseudomonas phage phiPSA2] (YP_009043273.1)
47	DNA packaging protein B	581	99	DNA packaging protein B [Pseudomonad phage gh-1] (NP_813786.1)
48	Hypothetical protein HP24	57	100	Hypothetical protein gh-1p42 [Pseudomonad phage gh-1] (NP_813788.1)

vB_PsaP-CHF 19 (MN729597)

ORF	Annotation	Protein Size (aa)	Identity with first Blastp hit protein (%)	Putative Function of first Blastp hit protein (Access number, NCBI)
1	Hypothetical protein HP1	84	99	Hypothetical protein WRT_001 [Pseudomonas virus WRT] (ARM69565.1)
2	Hypothetical protein HP2	66	67	Hypothetical protein PsPhHenninger_gp37 [Pseudomonas phage Henninger] (AUV61710.1)
3	Hypothetical protein HP3	103	93	Hypothetical protein PsPhHenninger_gp36 [Pseudomonas phage Henninger] (AUV61711.1)
4	Hypothetical protein HP4	159	99	Hypothetical protein phiPSA2_03 [Pseudomonas phage phiPSA2] (YP_009043231.1)

5	Hypothetical protein HP5	178	100	Hypothetical protein phiPSA2_04 [Pseudomonas phage phiPSA2] (YP_009043232.1)
6	Hypothetical protein HP6	234	100	Hypothetical protein phiPSA2_05 [Pseudomonas phage phiPSA2] (YP_009043233.1)
**7**	Hypothetical protein HP7	86	41	Hypothetical protein PsPhHenninger_gp32 [Pseudomonas phage Henninger] (AUV61715.1)
8	Hypothetical protein HP8	175	32	Hypothetical protein PsPhHenninger_gp31 [Pseudomonas phage Henninger] (AUV61716.1)
9	RNA polymerase	885	98	DNA-directed RNA polymerase [Pseudomonas phage phiPsa17] (AKG94352.1)
10	Hypothetical protein HP9	133	47	HNH endonuclease [Pseudomonas fluorescens] (WP_080547111.1)
11	Hypothetical protein HP10	133	100	Hypothetical protein gh-1p04 [Pseudomonad phage gh-1] (NP_813750.1)
12	DNA ligase	354	99	DNA ligase [Pseudomonas phage phiPsa17] (AKG94356.1)
13	Hypothetical protein HP11	93	98	Hypothetical protein phiPSA2_12 [Pseudomonas phage phiPSA2] (YP_009043240.1)
14	Hypothetical protein HP12	85	100	Hypothetical protein phiPSA2_13 [Pseudomonas phage phiPSA2] (YP_009043241.1)
15	Hypothetical protein HP13	215	100	Hypothetical protein gh-1p07 [Pseudomonad phage gh-1] (NP_813753.1)
16	RNA inhibitor	55	100	Putative host RNA-polymerase inhibitor [Pseudomonad phage gh-1] (NP_813754.1)
17	Hypothetical protein HP14	121	100	Hypothetical protein phiPSA2_16 [Pseudomonas phage phiPSA2] (YP_009043244.1)

18	ssDNA binding protein	233	100	Single-stranded DNA-binding protein [Pseudomonad phage gh-1] (NP_813756.1)
19	Endonuclease	147	100	Endonuclease I [Pseudomonad phage gh-1] (NP_813757.1)
20	Lyzozyme	146	99	Lysozyme [Pseudomonad phage gh-1] (NP_813758.1)
21	Hypothetical protein HP15	179	99	Hypothetical protein phiPSA2_20 [Pseudomonas phage phiPSA2] (YP_009043248.1)
22	Primase/Helicase	563	99	Primase/helicase protein [Pseudomonas phage phiPsa17] (AKG94365.1)
23	Hypothetical protein HP16	67	98	Hypothetical protein phiPSA2_22 [Pseudomonas phage phiPSA2] (YP_009043250.1)
24	Hypothetical protein HP17	169	100	Hypothetical protein gh-1p17 [Pseudomonad phage gh-1] (NP_813763.1)
25	DNA polymerase	715	100	DNA polymerase [Pseudomonas phage phiPsa17] (AKG94368.1)
26	Hypothetical protein HP18	127	100	Hypothetical protein gh-1p19 [Pseudomonad phage gh-1] (NP_813765.1)
27	Hypothetical protein HP19	69	100	Hypothetical protein gh-1p20 [Pseudomonad phage gh-1] (NP_813766.1)
28	Exonuclease	314	100	Exonuclease [Pseudomonad phage gh-1] (NP_813767.1)
29	Hypothetical protein HP20	80	100	Hypothetical protein gh-1p22 [Pseudomonad phage gh-1] (NP_813768.1)
30	Hypothetical protein HP21	90	100	Hypothetical protein phiPSA2_29 [Pseudomonas phage phiPSA2] (YP_009043257.1)
31	Hypothetical protein HP22	147	100	Hypothetical protein phiPSA2_30 [Pseudomonas phage phiPSA2]

				(YP_009043258.1)
32	Tail assembly	100	100	Tail assembly protein [Pseudomonas phage phiPSA2] (YP_009043259.1)
33	Head-tail connector protein	543	100	Head-tail connector protein [Pseudomonad phage gh-1] (NP_813772.1)
34	Capsid assembly protein	291	100	Capsid assembly protein [Pseudomonad phage gh-1] (NP_813773.1)
35	Major capsid protein	347	100	Major capsid protein [Pseudomonad phage gh-1] (NP_813774.1)
36	Tail tubular protein A	195	100	Tail tubular protein A [Pseudomonad phage gh-1] (NP_813775.1)
37	Tail fiber protein	718	99	Tail fiber protein [Pseudomonas phage phiPsa17] (AKG94380.1)
38	Internal virion protein A	144	100	Internal virion protein A [Pseudomonas phage phiPSA2] (YP_009043265.1)
39	Internal virion protein B	193	100	Internal virion protein B [Pseudomonad phage gh-1] (NP_813778.1)
40	Internal virion C	738	99	Internal virion protein C [Pseudomonas phage phiPSA2] (YP_009043267.1)
41	Internal virion D	1392	99	Internal virion protein D [Pseudomonad phage gh-1] (NP_813780.1)
42	Tail fiber protein	609	99	Tail fiber protein [Pseudomonas phage phiPSA2] (YP_009043269.1)
43	Hypothetical protein HP23	118	100	Hypothetical protein phiPSA2_42 [Pseudomonas phage phiPSA2] (YP_009043270.1)
44	Holin	71	100	Type II holing [Pseudomonas phage phiPSA2]

				(YP_009043271.1)
45	DNA packaging protein	85	100	DNA packaging protein, small subunit [Pseudomonad phage gh-1] (NP_813784.1)
46	Lysin	149	99	Rz-like lysis protein [Pseudomonas phage phiPSA2] (YP_009043273.1)
47	DNA packaging protein B	581	100	DNA packaging protein B [Pseudomonad phage gh-1] (NP_813786.1)
48	Hypothetical protein HP24	57	100	Hypothetical protein gh-1p42 [Pseudomonad phage gh-1] (NP_813788.1)

vB_PsaP-CHF 21 (MN729598)

ORF	Annotation	Protein Size (aa)	Identity with first Blastp hit protein (%)	Putative Function of first Blastp hit protein (Access number, NCBI)
1	Hypothetical protein HP1	84	99	Hypothetical protein WRT_001 [Pseudomonas virus WRT] (ARM69565.1)
2	Hypothetical protein HP2	66	67	Hypothetical protein PsPhHenninger_gp37 [Pseudomonas phage Henninger] (AUV61710.1)
3	Hypothetical protein HP3	103	93	Hypothetical protein PsPhHenninger_gp36 [Pseudomonas phage Henninger] (AUV61711.1)
4	Hypothetical protein HP4	159	99	Hypothetical protein phiPSA2_03 [Pseudomonas phage phiPSA2] (YP_009043231.1)
5	Hypothetical protein HP5	178	100	Hypothetical protein phiPSA2_04 [Pseudomonas phage phiPSA2] (YP_009043232.1)
	Hypothetical protein			Hypothetical protein phiPSA2_05 [Pseudomonas phage phiPSA2]

6	HP6	234	100	(YP_009043233.1)
**7**	Hypothetical protein HP7	86	38	Hypothetical protein PsPhHenninger_gp32 [Pseudomonas phage Henninger] (AUV61715.1)
8	Hypothetical protein HP8	146	99	Hypothetical protein phiPSA2_06 [Pseudomonas phage phiPSA2] (YP_009043234.1)
9	RNA polymerase	885	100	DNA-directed RNA polymerase [Pseudomonas phage phiPsa17] (AKG94352.1)
10	Hypothetical protein HP9	90	99	Hypothetical protein gh-1p03 [Pseudomonad phage gh-1] (WP_080547111.1)
11	Hypothetical protein HP10	124	89	Hypothetical protein gh-1p04 [Pseudomonad phage gh-1] (NP_813750.1)
12	DNA ligase	354	99	DNA ligase [Pseudomonas phage phiPsa17] (AKG94356.1)
13	Hypothetical protein HP11	93	99	Hypothetical protein phiPSA2_12 [Pseudomonas phage phiPSA2] (YP_009043240.1)
14	Hypothetical protein HP12	85	100	Hypothetical protein phiPSA2_13 [Pseudomonas phage phiPSA2] (YP_009043241.1)
15	Hypothetical protein HP13	215	100	Hypothetical protein gh-1p07 [Pseudomonad phage gh-1] (NP_813753.1)
16	RNA inhibitor	55	100	Putative host RNA-polymerase inhibitor [Pseudomonad phage gh-1] (NP_813754.1)
17	Hypothetical protein HP14	121	100	Hypothetical protein phiPSA2_16 [Pseudomonas phage phiPSA2] (YP_009043244.1)
18	ssDNA binding protein	233	100	Single-stranded DNA-binding protein [Pseudomonad phage gh-1] (NP_813756.1)
19	Endonuclease	147	100	Endonuclease I [Pseudomonad phage gh-1] (NP_813757.1)

20	Lyzozyme	146	99	Lysozyme [Pseudomonad phage gh-1] (NP_813758.1)
21	Hypothetical protein HP15	179	99	Hypothetical protein phiPSA2_20 [Pseudomonas phage phiPSA2] (YP_009043248.1)
22	Primase/Helicase	563	99	Primase/helicase protein [Pseudomonas phage phiPsa17] (AKG94365.1)
23	Hypothetical protein HP16	67	100	Hypothetical protein phiPSA2_22 [Pseudomonas phage phiPSA2] (YP_009043250.1)
24	Hypothetical protein HP17	169	100	Hypothetical protein gh-1p17 [Pseudomonad phage gh-1] (NP_813763.1)
25	DNA polymerase	715	100	DNA polymerase [Pseudomonas phage phiPsa17] (AKG94368.1)
26	Hypothetical protein HP18	127	99	Hypothetical protein gh-1p19 [Pseudomonad phage gh-1] (NP_813765.1)
27	Hypothetical protein HP19	69	98	Hypothetical protein gh-1p20 [Pseudomonad phage gh-1] (NP_813766.1)
28	Exonuclease	314	100	Exonuclease [Pseudomonad phage gh-1] (NP_813767.1)
29	Hypothetical protein HP20	80	100	Hypothetical protein gh-1p22 [Pseudomonad phage gh-1] (NP_813768.1)
30	Hypothetical protein HP21	90	100	Hypothetical protein phiPSA2_29 [Pseudomonas phage phiPSA2] (YP_009043257.1)
31	Hypothetical protein HP22	147	100	Hypothetical protein phiPSA2_30 [Pseudomonas phage phiPSA2] (YP_009043258.1)
32	Tail assembly	100	100	Tail assembly protein [Pseudomonas phage phiPSA2] (YP_009043259.1)
33	Head-tail connector protein	543	99	Head-tail connector protein [Pseudomonad phage gh-1]

				(NP_813772.1)
34	Capsid assembly protein	291	100	Capsid assembly protein [Pseudomonad phage gh-1] (NP_813773.1)
35	Major capsid protein	347	100	Major capsid protein [Pseudomonad phage gh-1] (NP_813774.1)
36	Tail tubular protein A	195	100	Tail tubular protein A [Pseudomonad phage gh-1] (NP_813775.1)
37	Tail fiber protein	718	99	Tail fiber protein [Pseudomonas phage phiPsa17] (AKG94380.1)
38	Internal virion protein A	144	100	Internal virion protein A [Pseudomonas phage phiPSA2] (YP_009043265.1)
39	Internal virion protein B	193	99	Internal virion protein B [Pseudomonad phage gh-1] (NP_813778.1)
40	Internal virion C	738	99	Internal virion protein C [Pseudomonas phage phiPSA2] (YP_009043267.1)
41	Internal virion D	1392	96	Internal virion protein D [Pseudomonad phage gh-1] (NP_813780.1)
42	Tail fiber protein	609	98	Tail fiber protein [Pseudomonas phage phiPSA2] (YP_009043269.1)
43	Hypothetical protein HP23	118	100	Hypothetical protein phiPSA2_42 [Pseudomonas phage phiPSA2] (YP_009043270.1)
44	Holin	71	84	Type II holing [Pseudomonas phage phiPSA2] (YP_009043271.1)
45	DNA packaging protein	85	100	DNA packaging protein, small subunit [Pseudomonad phage gh-1] (NP_813784.1)
46	Lysin	149	99	Rz-like lysis protein [Pseudomonas phage phiPSA2] (YP_009043273.1)
	DNA packaging			DNA packaging protein B

47	protein B	581	100	[Pseudomonad phage gh-1] (NP_813786.1)
48	Hypothetical protein HP24	57	100	Hypothetical protein gh-1p42 [Pseudomonad phage gh-1] (NP_813788.1)

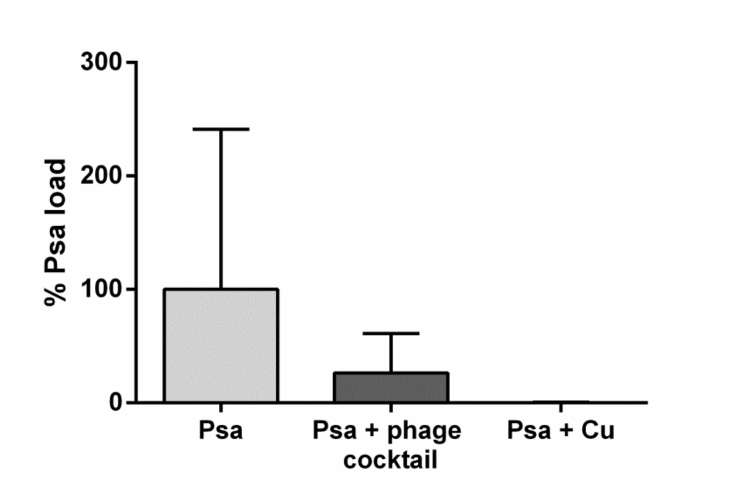

Supplementary figure 2. **Psa load on kiwifruit leaves after a 30 days assay with different treatments**. The results represent the average of two independent assays with five replicates each (25 leaves per assay). Psa load in leaves of plants without antimicrobial treatment was considered as 100%. The phage cocktail corresponds to equal amounts of the phages CHF1, CHF7, CHF19 and CHF21. The bacteria were inoculated in plants at day one of the assay. The phage cocktail and copper treatments were added 1-hour post infection with the bacteria at day 1 of the assay.
